# Insomnische Symptome und Suizidalität – Zusammenhänge und Management

**DOI:** 10.1007/s40211-023-00466-z

**Published:** 2023-05-12

**Authors:** Dirk Schwerthöffer, Hans Förstl

**Affiliations:** Klinik und Poliklinik für Psychiatrie und Psychotherapie, TU-München, Ismaningerstraße 22, 81675 München, Deutschland

**Keywords:** Insomnische Symptome, Insomnie, Suizidalität, Risikofaktoren für Suizidalität, Schlafstörungen, Hypofrontalität, Symptoms of insomnia, Insomnia, Suicidality, Risk factors for Suicidality, Sleep disturbances, Hypofrontality

## Abstract

**Hintergrund:**

Ein Zusammenhang zwischen insomnischen Symptomen und Suizidalität wurde lange vermutet und ist von besonderem klinischem Interesse.

**Ziel:**

Wir untersuchen aktuelle Hinweise aus Epidemiologie und Neurobiologie auf diesen Zusammenhang, um ein gezieltes Management vorzuschlagen.

**Material und Methode:**

Klinisches Beispiel und selektive Medline-Literaturrecherche zu insomnischen Symptomen und Suizidalität.

**Ergebnisse:**

Epidemiologische Daten weisen auf insomnische Symptome als unabhängigen Risikofaktor für Suizidalität hin. Neurobiologische Befunde unterlegen eine Beziehung zwischen insomnischen Symptomen und Suizidalität, u. a. durch eine serotonerge Dysfunktion sowie eine besonders beeinträchtigte circadiane Rhythmik mit konsekutiver Hypofrontalität, beeinträchtigter Problemlösefähigkeit und verminderter Impulskontrolle. Im Rahmen der Suizidprävention muss bei Patienten mit kombinierten insomnischen und depressiven Symptomen nachdrücklich nach weiteren Risikofaktoren für Suizidalität gesucht werden, u. a. soziale Isolation, nächtliche Grübelneigung, komorbide psychische Erkrankungen, Zugang zu potenziell toxischen Pharmaka oder Waffen.

**Schlussfolgerung:**

Besonders bei Patienten mit weiteren Suizid-Risikofaktoren müssen insomnische Symptome frühzeitig konsequent behandelt werden. In der Pharmakotherapie sind für Patienten mit insomnischen Symptomen und Suizidalität schlaffördernde Antidepressiva mit niedriger Toxizität und Antipsychotika vorzuziehen. Eine an den circadianen Rhythmus angepasste multimodale antiinsomnische und antidepressive Therapie könnte die Zusammenhänge zwischen depressiv-suizidalen und insomnischen Symptomen günstig beeinflussen.

## Abstract (en)

**Background:**

A link between insomniac symptoms and suicidality has long been suspected and deserves specific attention.

**Objective:**

We examine the current evidence for this relationship from epidemiology and neurobiology in order to propose a targeted management.

**Material and method:**

Clinical example and selective Medline-literature research for insomnia symptoms and suicidality.

**Results:**

Epidemiological data and statistical analysis show that symptoms of insomnia are independent risk factors for suicidality. Neurobiological factors associated with combined insomnia symptoms and suicidality are: serotonergic dysfunction and circadian rhythm disorder leading to hypofrontality with reduced problem solving capacity and impaired emotional and impulse-control. Social isolation, recurrent rumination, comorbid psychiatric disorders, access to potentially lethal drugs or weapons need urgent evaluation in patients with a combination of suicidality and symptoms of insomnia.

**Conclusion:**

patients with insomnia and further risk factors for suicide need to be treated resolutely and at an early stage. Modern sleep-promoting antidepressants with low toxicity and antipsychotics must be preferred in the treatment of patients with insomniac sleep disorders and suicidality. Multimodal anti-insomnia and anti-depressive therapy adapted to the circadian rhythm can exert a favorable influence both on depressive-suicidal and insomnia symptoms and their inherent risks.

## Einleitung

1914 vermutete C. Ernest Pronger, dass eine große Anzahl von Suiziden im Zusammenhang mit Insomnie stünde und deshalb größtenteils verhindert werden könnte [56]. Er beklagte fehlendes Wissen über die Ursachen der Insomnie und entsprechende Behandlungsmöglichkeiten. Inzwischen weisen zahlreiche epidemiologische Daten auf den Zusammenhang zwischen insomnischen Symptomen und Suizidalität hin. Nach einer Kasuistik und der Darstellung epidemiologischer Daten führen wir einige neurobiologische Überlegungen zu Art und Ursache dieses Zusammenhangs auf.

## Begriffsbestimmung

In Klinik und Literatur werden die Begriffe „Insomnie“, „insomnische Symptomen“, „insomnische Schlafstörungen“, „Schlaflosigkeit“ und „symptomatische Schlaflosigkeit“ oft nur unscharf voneinander abgegrenzt. Insomnische Symptome wie z. B. verkürzte Schlafdauer, Einschlafstörung oder Früherwachen müssen von der Diagnose einer „Insomnie“ abgegrenzt werden. Die Diagnosekritierien für eine Insomnie umfassen neben Ein‑, Durchschlafstörungen und Früherwachen von entsprechender Dauer und Häufigkeit auch das subjektive Empfinden einer Schlafstörung mit gestörtem Wohlbefinden am Tage (International Classification of Sleep Disorders) und sind sowohl von objektiv gestörtem Schlaf, als auch von verkürzter Schlafdauer oder Schlafentzug abzugrenzen.

Diese Arbeit behandelt überwiegend den Zusammenhang zwischen „insomnischen Symptomen“ und Suizidalität.

## Kasuistik

Ein 60-jähriger Finanzmanager wird um 3:00 Uhr morgens via Notarzt mit einer selbst zugefügten, thorakalen Schnittverletzung stationär aufgenommen, zunächst wegen eines Hämatothorax viszeralchirurgisch versorgt und nach 3‑tägiger intensivmedizinischer Behandlung in die Klinik für Psychiatrie übernommen. Es bestehen keine relevanten körperlichen Vorerkrankungen und es fanden keine psychotherapeutischen oder psychiatrischen Vorbehandlungen statt. Die Nachbarin des Patienten war durch einen lauten Sturz in seiner Wohnung alarmiert worden und hatte, nachdem er nicht auf ihr Klingeln reagierte, den Rettungsdienst verständigt. Im blutverschmierten Wohnzimmer des Patienten werden zwei leere Tablettenblister für insgesamt 20 Tabletten Zolpidem 10 mg aufgefunden. Bei der Aufnahme in die psychiatrische Klinik ist der Patient deutlich niedergestimmt, kaum schwingungsfähig und psychomotorisch verlangsamt. Er berichtet über eine Amnesie für die Ereignisse am Abend der Klinikeinweisung. Er sei, wie immer in den letzten Wochen, „ganz früh schlafen gegangen um genug Ruhe und Erholung für den Folgetag zu finden“. Seit mehreren Monaten bestünde bereits eine massive berufliche Überforderungssituation durch gleichzeitige Leitungsverantwortung für zwei Projekte. Nach und nach habe das bei ihm zu ständiger Erschöpfung, Lust- und Freudlosigkeit und Vernachlässigung fast aller Freizeitaktivitäten und sozialer Kontakte geführt. Der Patient habe „nur noch gearbeitet“, sich „ansonsten vollkommen isoliert“ und sei zuletzt „emotional abgestumpft“. Sein Leben habe „nur noch aus 16 h Büro und 8 h Schlaf“ bestanden. Am Ende sei er so kraftlos gewesen, dass er für die einfachsten Dinge des Alltags keinen Antrieb mehr gefunden habe. Umso wichtiger sei es ihm gewesen, immer „mindestens acht Stunden lang zu schlafen um so den nächsten Tag durchzustehen“. Häufig habe er aber nachts wachgelegen und sei in Gedanken, die Termine des Folgetags durchgegangen. Seit acht Wochen nehme er nun wegen einer hausärztlich diagnostizierten Insomnie mit führender Durchschlafstörung und eingeschränkter Tagesbefindlichkeit, allabendlich Zolpidem 10 mg/d ein. Suizidgedanken seien dann, erstmals in seinem Leben, vor zwei Wochen in Verbindung mit der Sorge, „nie wieder richtig schlafen zu können“ aufgetreten und hätten sich in den letzten drei Tagen, in Verbindung mit ständiger innerer Unruhe bei gleichzeitig bestehender starker Erschöpfung, massiv verstärkt. Daraufhin habe der Patient die Zolpidem-Dosierung von zwei auf fünf Tabletten täglich gesteigert um „endlich Ruhe zu finden“.

## Epidemiologie

Weltweit sterben annähernd 800.000 Menschen jährlich durch Suizid, davon knapp 10.000 in Deutschland und etwa 1000 in Österreich [25]. Suizidalität ist ein vielschichtiges Gesundheitsproblem mit zahlreichen psychosozialen aber auch neurobiologischen Risikofaktoren, wie z. B. affektiven Erkrankungen, Suchterkrankungen, hohem Alter und Zugang zu Schusswaffen [72]. Auf der Suche nach weiteren, möglichst beeinflussbaren Risikofaktoren, wurde in den letzten Jahren verstärkt der Einfluss insomnischer Symptome auf Suizidalität untersucht [22]. 10–35 % der erwachsenen Bevölkerung ist von klinisch relevanten Schlafstörungen betroffen [45, 62], darunter gehäuft Menschen mit psychischen Erkrankungen.

Die Abgrenzung des Einflusses depressiver Erkrankungen auf die Beziehung von Insomnie und Suizidalität fällt oft schwer, da sowohl insomnische Symptome als auch Suizidalität Kernsymptome schwerer Depressionen sind und selber in bidirektionaler Verbindung zueinander stehen. So treten insomnische Symptome gehäuft im Rahmen von depressiv-suizidalen Episode auf, wirken aber auch für sich depressiogen. In diesem Zusammenhang wären Studien über den Zusammenhang zwischen insomnischen Symptomen und Suizidalität mit depressiven Störungen als Ausschlusskriterium von Interesse, die unserer Kenntnis nach aber nicht vorliegen.

Die Metaanalyse von Harris [22] fand 42 geeignete Studien, die den Zusammenhang zwischen Schlafstörungen und Suizidalität untersuchen und bis dato liegen acht systematische Reviews (davon fünf Metaanalysen) dazu vor [4, 11, 22, 26, 32, 33, 35, 53]. Da diesen Arbeiten überwiegend retrospektive Studien und Querschnittsanalysen zugrunde liegen und prospektive und longitudinale Untersuchungen weitgehend fehlen, ist von einer „moderaten empirischen Grundlage“ für den Zusammenhang zwischen Schlafstörungen und Suizidalität auszugehen [33]. Schlafstörungen werden von einigen Autoren als statistisch schwach signifikanter, aber unabhängiger Risikofaktor für Suizidalität angesehen, mit den stärksten Zusammenhängen für Insomnie und Alpträume [22].

Die US-amerikanische „National Comorbidity Survey Replication“ zeigte, dass in der Allgemeinbevölkerung, unabhängig von psychischen Erkrankungen, Ein- und Durchschlafstörungen sowie morgendliches Früherwachen mit einem bis zu zehnfach erhöhten Risiko verbunden sind, innerhalb eines Jahres Suizidalität zu entwickeln [74]. Eine aktuelle prospektive Kohortenstudie wies nach, dass Insomnie in Verbindung mit kurzer Nachtschlafdauer (< 7 h) das Suizidrisiko erhöht [23]. Gleichzeitig sind in diesem Zusammenhang auch Qualität und Ausprägungsgrad insomnischer Beschwerden von Bedeutung. Eine große Kohortenstudie ergab, dass Männer mit drei oder mehr Merkmalen eines beeinträchtigten Nachtschlafs (Schlaftabletteneinnahme, Einschlafstörung, nächtliches Wachliegen, Früherwachen, subjektiv schlechter Nachtschlaf) ein nahezu fünffach erhöhtes Suizidrisiko aufwiesen [58]. In mehreren Untersuchungen wurde festgestellt, dass fast alle Patienten im Vorfeld schwerer Suizidversuche Ärzte aufsuchen, dort aber vorwiegend über Schlafstörungen und nicht über Suizidgedanken klagen [27]. Passend dazu gaben in einer schwedischen Querschnittsstudie 89 % aller Patienten nach Suizidversuchen an, im Vorfeld unter Insomnie und Albträumen gelitten zu haben [65]. Für manche Populationen, wie z. B. Patienten mit psychischen Komorbiditäten im Allgemeinen [64], Erkrankungen aus dem schizophrenen Formenkreis im Speziellen [43] oder Militärveteranen [71] besteht ein besonders hohes Risiko beim Vorliegen insomnischer Symptome suizidal zu werden. Eine große Fragebogenuntersuchung an 583 US-amerikanischen College-Studenten erbrachte, dass sowohl Alpträume als auch insomnische Symptome, unabhängig voneinander mit Suizidgedanken verbunden sind [47]. Bei Patienten mit körperlichen Ursachen für insomnische Symptome, wie z. B. Schmerzsyndromen [2] und dem Restless-Legs-Syndrom [10] tritt Suizidalität ebenfalls gehäuft auf.

## Neurobiologische Befunde

### Circadiane Rhythmik

Nicht nur zeitliche Aspekte [14], sondern auch biologische circadiane Rhythmen determinieren Suizidalität. Nächtliches Wachsein ist mit vermehrten Suizidgedanken [69] und bei US-Veteranen mit vermehrten Suiziden assoziiert [40]. Männliche Schichtarbeiter leiden vermehrt unter Suizidgedanken [50] und nächtliche Lichtexposition wird mit vermehrten Suizidhandlungen in Verbindung gebracht [44]. Zirkadiane Abendtypen („Eulen“) weisen eine stärkere Impulsivität und eine größere Bereitschaft für gewaltsame Suizidversuche auf als Neutral- oder Morgentypen [60]. Dabei können ein verstärktes nächtliches Gefühl der Isolation und vermehrter Alkohol- und Substanzabusus in den Nachtstunden eine Rolle spielen und besonders für Menschen mit Zugang zu Waffen zu einem Risiko werden [51].

Grundsätzlich bedingt die circadiane Rhythmik eine nächtliche *Hypoaktivität des präfrontalen Kortex* mit entsprechend abgeschwächter Kontrolle von Impulsen und Affekten, deren Entstehung überwiegend mit subkortikalen Hirnstrukturen wie dem meso-limbischen System und den Basalganglien in Verbindung gebracht werden. Dies kann, besonders bei gleichzeitig bestehenden insomnischen und depressiven Symptomen und in Verbindung mit enthemmenden Substanzen (wie Alkohol oder Benzodiazepin-Hypnotika) zu verminderter rationaler Problemlösefähigkeit und vermehrter gewalttätiger Impulsivität führen und so die Schwelle für suizidales Verhalten senken [70].

### Bildgebung und Neurophysiologie

Patienten die gleichzeitig unter schweren depressiven und insomnischen Symptomen leiden, zeigen eine kortikale Hirnvolumenminderung der grauen Substanz [29, 50], Veränderungen des Hirnstoffwechsels (u. a. in den Bereichen Amygdala, und präfrontaler Kortex) [3] und eine veränderte Konnektivität in kortikalen und meso-limbischen Strukturen (u. a. lateraler orbitofrontaler Kortex, cingulärer Kortex, Amygdala, Nucleus accumbens) [20].

Zudem zeigen Patienten mit suizidalen Syndromen in verschiedenen Untersuchungen verschiedene* neurophysiologische Korrelate* gestörten Schlafs. Dazu gehören Veränderungen der Schlafarchitektur (verlängerte Einschlaf- und REM-Latenz), erhöhter Anteil des Schlafstadiums NREM1, vermehrte REM-Dichte [6] und polysomnographisch messbare Hyperarousals im Schlaf [16]. Des weiteren berichten Patienten mit gleichzeitiger Suizidalität und insomnischen Symptomen oft über kognitive Arousals in der Einschlafphase mit intrusiven Kognitionen oder vegetativer Erregung [21].

### Neurochemie

Sowohl bei insomnischen als auch bei depressiv-suizidalen Symptomen wird eine* serotonerge Dysfunktion *vermutet. *Serotonin* moduliert gleichzeitig Affekt und circadiane Rhythmen [55], supprimiert den REM-Schlaf und unterliegt in seiner Aktivität selber dem circadianen Schlaf-Wach-Zyklus mit niedrigsten Konzentrationen während des Tiefschlafs und REM-Schlafs. Die schnell einsetzenden antidepressiven Effekte eines therapeutischen Schlafentzugs stehen in vollkommenem Gegensatz zur depressiogenen Wirkung einer chronischen Insomnie und werden u. a. durch einen erhöhten Serotonin-Turnover und Anstieg des Bindungspotentials des *Serotonin*-Rezeptors 5‑HT_2A_R in mehreren Hirnregionen erklärt [17]. Patienten nach schweren Suizidversuchen wiederum weisen erniedrigte Serotonin-Konzentrationen im Liquor [15], eine verminderte Serotonin-Aktivität im präfrontalen Kortex [30] und eine erhöhte REM-Schlaf-Dauer und -Dichte auf.

Eine weitere Gemeinsamkeit depressiver Störungen und insomnischer Symptome ist eine Dysregulation der HPA-Achse (hypothalamic-pituitary-adrenal axis) [1]. Beide Störungsbilder werden mit einer verminderten nächtlichen Cortisolabsenkung und verminderter nächtlicher Monoamin-Suppression [41, 48] in Verbindung gebracht. Cortisol verhindert Schlaf und ist sowohl im Serum von Patienten mit depressiv-suizidalen Syndromen als auch nach Suizidversuchen erhöht [37]. Gleichzeitig unterliegt die Cortisol-Konzentration selber einer circadianen Rhythmik mit frühmorgendlichem Maximum und Absinken im Tagesverlauf. Eine gestörte Cortisol-Ausschüttung führt unter anderem zu einer Dysregulation des Tiefschlaf-induzierenden Neuropeptids *DSIP *(delta-sleep inducing peptide) [49].

**Figure 1:**
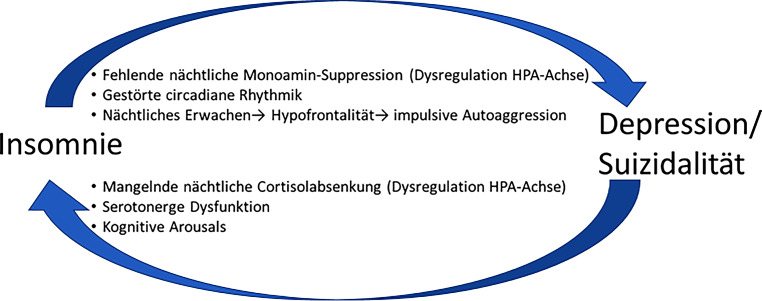

Abb. 1 fasst einige mögliche neurobiologische Verbindungen zwischen insomnischen und depressiv-suizidalen Symptomen zusammen.

## Prävention und Behandlung

Evidenzbasiert wirksame Maßnahmen in der Suizidprävention sind unter anderem: Schulungen und Aufklärungskampagnen, die Behandlung psychischer Erkrankungen und die Beschränkung des Zugangs zu Tötungsmethoden wie Schusswaffen und Suizidhotspots [36]. Da insomnische Symptome zu Suizidalität beitragen, sollte ihre Erfassung, als potenziell suizidpräventive Maßnahmen [46] in das Screening von Suizid-gefährdeten Patienten einbezogen und im Kontext mit anderen Risikofaktoren bewertet werden. Besonders nach vorausgegangenen Suizidversuchen oder beim Vorliegen weiterer Risikofaktoren müssen schlafmedizinische Interventionen in die Primär- und Sekundärprävention miteinbezogen werden [5].

Wie bereits erwähnt, könnten insomnische Symptome Suizidalität direkt oder indirekt (z. B. über die Verstärkung einer Depression) beeinflussen. Zur genaueren Differenzierung sind Studien über den Einfluss schlaftherapeutischer Maßnahmen auf Suizidalität, unabhängig von ihrer Wirkung auf depressive Symptome [38], aufschlussreich. Sowohl für die Kognitive Verhaltenstherapie der Insomnie [67] als auch für eine schlaffördernde Pharmakotherapie [39] ließ sich eine solche, vom antidepressiven Effekt unabhängige, antisuizidale Wirkung zeigen.

### Nicht-pharmakologische Therapie

Die primäre Behandlung insomnischer Symptome erfolgt mittels Schlafhygiene, kognitiver Verhaltenstherapie und Pharmakotherapie. Für die Wahl der geeigneten Therapiemethode, ist es aufschlussreich, wie Patienten mit kombinierten insomnischen Symptomen und Suizidalität Schlaf und Lebensüberdruss in Verbindung bringen. In einer Interviewstudie bezeichnen viele Teilnehmer nächtliche Schlaflosigkeit als „riskant“, da sie dann weder ihre Familie noch ihre Freunde erreichen können. Schlaf wird auch von vielen Befragten als Alternative zu einem Suizid angesehen, indem er die Möglichkeit bietet „den Problemen des Alltags zu entkommen“ [31]. Schlaflosigkeit kann einem schwer depressiven Patienten also das Gefühl vermitteln, seinen letzten Zufluchtsort zu verlieren. Viele Patienten erklären Suizidversuche auch durch einen „Wunsch nach Ruhe“. Oft begegnen sie diesem Wunsch bereits frühzeitig durch dysfunktionales Verhalten, wie Schlafperioden tagsüber oder besonders frühe Bettzeiten, wodurch es aber nur zu einer Verstärkung des „insomnischen Teufelskreises“ mit vermehrtem Wachliegen, Grübeln, steigender Anspannung und Suizidalität kommt. Gleichzeitig berichten Patienten mit nächtlichem „nicht-konstruktivem Grübeln“ gehäuft über kombinierte insomnische Symptome und Suizidalität, oft auch im Zusammenhang mit Albträumen [18].

*Schlafhygienische Maßnahmen* und *Psychoedukation *fördern die Schlafqualität indem sie Schlafdauer, Schlafzeitpunkt, Schlafumgebung und körperliche Aktivität positiv beeinflussen und wirken auch nachgewiesen auf komorbide depressiv-suizidale Symptome [34].

Eine *kognitive Verhaltenstherapie *kann gleichzeitig Suizidalitäts-assoziierte Faktoren, wie Einsamkeit, Grübelneigung [59] oder Verzweiflung identifizieren und behandeln und die Schlafqualität verbessern [13]. Die spezifische Kognitive Verhaltenstherapie der Nicht-organischen Insomnie (CBT-I) konnte in einer Untersuchung an US-Veteranen, die unter insomnischen Symptomen litten, Suizidgedanken vermindern [67].

Aktuelle standardisierte, verhaltenstherapeutische Interventionen für Patienten nach einem Suizidversuch enthalten Maßnahmen zur Verbesserung des Schlafs [66] und auch Internet-basierte Psychotherapie-Verfahren könnten bei kombinierter Insomnie und Suizidalität wirksam sein [12].

### Pharmakotherapie

Die vorteilhafteste Behandlung für einen Patienten mit insomnischen Symptomen und komorbider Suizidalität sollte gleichermaßen zu einer Verbesserung der Schlafqualität und zu einem sofortigen Rückgang suizidaler Symptome führen. Eine mögliche antisuizidale Wirkung einer solchen Behandlung [39] muss aber gegen das Risiko der bekannten Häufung von Suiziden bei regelmäßigem Hypnotikagebrauch [8, 8] und vereinzelten Berichten über Suizidalität unter einer Psychopharmakotherapie [42] abgewogen werden.

Die gebräuchlichsten Schlafmittel sind Benzodiazepine und Benzodiazepin-Agonisten („Z-Substanzen“). Sie haben eine Zulassung für die Kurzzeitbehandlung der akuten Insomnie und könnten besonders bei Patienten mit schwerer Insomnie und Suizidgedanken antisuizidal wirksam sein [39]. Gleichzeitig weisen sie aber ein relevantes Missbrauchs- und Abhängigkeitspotential auf [24], sind mit einer bis zu vierfach erhöhten Mortalität assoziiert und können auch in suizidaler Absicht überdosiert werden [28].

Stattdessen sind schlaffördernde Antidepressiva aufgrund ihres fehlenden Missbrauchs- und Abhängigkeitspotentials, ihrer klaren Indikation bei depressiven Störungen und Hinweisen für ihre antisuizidale Wirkung bei Patienten mit kombinierter insomnischer und suizidaler Symptomatik [19] vorteilhafter. Dabei wirken einzelne schlaffördernde Antidepressiva (wie z. B. Mirtazapin, Trazodon oder Doxepin) sehr unterschiedlich auf die Schlafarchitektur und werden bei insomnischen Symptomen auch häufig in niedriger (subtherapeutisch antidepressiver) Dosis verordnet. Sie werden aber in aktuellen Insomnie-Leitlinien, bei fehlenden Evidenzen, nur eingeschränkt für die Pharmakotherapie der Insomnie empfohlen.

Die Verordnung sedierender Antipsychotika, wie z. B. Quetiapin, zum Schlafanstoß ist weitverbreitet und scheint ebenfalls insomnische und komorbide depressive Symptome verbessern zu können [52]. Zusätzlich liegen für einzelne Antipsychotika Hinweise für ihren antisuizidalen Effekt mit und ohne komorbider Insomnie vor [54]. Dennoch wird ihr Einsatz als Hypnotika, aufgrund ihres antipsychotischen Nebenwirkungsprofils, von vielen Klinikern und Autoren kritisch bewertet [7].

## Multimodale Therapie von insomnischen Symptomen und komorbider Suizidalität im circadianen Rhythmus

Obwohl chronotherapeutische und chronopharmakologische Maßnahmen bereits in vielen medizinischen Bereichen angewendet werden, liegen kaum praktische Empfehlungen zur Implementierung circadianer Therapien in die „breitere klinische Praxis“ der Depressionsbehandlung vor [57].

**Figure 2:**
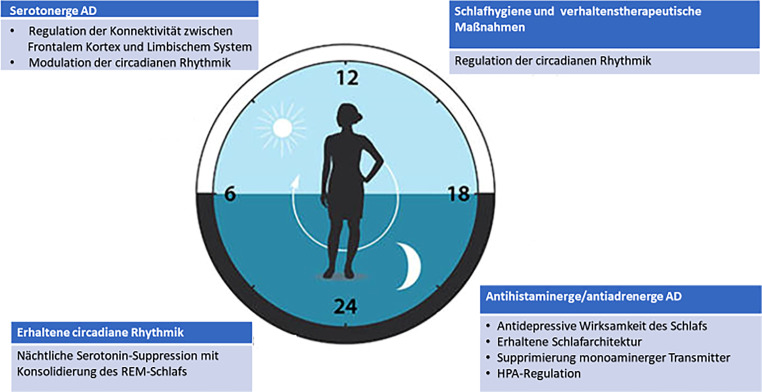

Abb. 2 fasst einige modellhafte Überlegungen und Vorschläge zu einer an den circadianen Rhythmus angepassten multimodalen Therapie von komorbiden insomnischen und depressiv-suizidalen Symptomen zusammen, die neurobiologische und chronobiologische Aspekte berücksichtigt.

*Vormittags* eingenomme serotonerge Antidepressiva entwickeln ihre höchste Serum-Konzentration tagsüber, stören so, bei sinkender abendlicher Konzentration weniger ausgeprägt die nächtliche Schlafarchitektur, wirken über eine Regulation der fronto-limbischen Konnektivität [9] nächtlicher Hypofrontalität entgegen und modulieren circadiane Rhythmen [63].

*Tagsüber* durchgeführte schlafhygienische Maßnahmen, wie z. B. reduzierte Bettzeiten, regulieren die circadiane Rhythmik.

Die *abendliche* Einnahme antihistaminerg und antiadrenerg wirkender Antidepressiva reguliert die HPA-Achse, supprimiert monoaminerge Transmitter und verstärkt durch die sedierende Komponente die antidepressive Wirksamkeit des Nachtschlafs [61, 73].

## Fazit

1. Besonders bei Patienten mit weiteren Risikofaktoren für Suizidalität müssen insomnische Symptome frühzeitig erkannt und konsequent behandelt werden.
2. Schlaffördernde Antidepressiva stellen für Patienten mit insomnischen Symptomen und Suizidalität eine vorteilhafte Pharmakotherapie dar.
3. Eine an den circadianen Rhythmus angepasste multimodale Therapie könnte die Zusammenhänge von kombinierten insomnischen und depressiv-suizidalen Symptomen günstig beeinflussen.
